# Positron annihilation localization by nanoscale magnetization

**DOI:** 10.1038/s41598-020-76980-9

**Published:** 2020-11-20

**Authors:** Yaser H. Gholami, Hushan Yuan, Moses Q. Wilks, Lee Josephson, Georges El Fakhri, Marc D. Normandin, Zdenka Kuncic

**Affiliations:** 1 Faculty of Science, School of Physics, The University of Sydney, Sydney, NSW Australia; 2 Sydney Vital Translational Cancer Research Centre, St Leonards, NSW Australia; 3 Bill Walsh Translational Cancer Research Laboratory, The Kolling Institute, Northern Sydney Local Health District, Sydney, Australia; 4 Gordon Center for Medical Imaging, Harvard Medical School, Massachusetts General Hospital, Boston, MA USA; 5 The University of Sydney Nano Institute, Sydney, NSW Australia

**Keywords:** Biological physics, Nanomedicine, Diagnostic devices, Imaging techniques and agents, Nanotechnology in cancer

## Abstract

In positron emission tomography (PET), the finite range over which positrons travel before annihilating with an electron places a fundamental physical limit on the spatial resolution of PET images. After annihilation, the photon pair detected by the PET instrumentation is emitted from a location that is different from the positron-emitting source, resulting in image blurring. Here, we report on the localization of positron range, and hence annihilation quanta, by strong nanoscale magnetization of superparamagnetic iron oxide nanoparticles (SPIONs) in PET-MRI. We found that positron annihilations localize within a region of interest by up to 60% more when SPIONs are present (with [Fe] = 3 mM) compared to when they are not. The resulting full width at half maximum of the PET scans showed the spatial resolution improved by up to $\approx$ 30%. We also found evidence suggesting that the radiolabeled SPIONs produced up to a six-fold increase in ortho-positronium. These results may also have implications for emerging cancer theranostic strategies, where charged particles are used as therapeutic as well as diagnostic agents and improved dose localization within a tumor is a determinant of better treatment outcomes.

## Introduction

Positron Emission Tomography (PET) is an essential medical imaging technology for detecting and diagnosing diseases such as cancer and Alzheimer’s^1,2^. It works by detecting pairs of photons emitted following electron–positron annihilation inside the body after administration of a positron-emitting radiopharmaceutical. Although PET is a widely available clinical technology, its accuracy is limited by the spread in positron range, the distance a positron travels before it annihilates. This imposes a fundamental physical limit on the spatial resolution of PET images, a problem that has been studied for many years^1, 3–5^. The spatial resolution of PET imaging is also limited by lack of collinearity of the annihilation photons and other instrument related factors such as detector size and material, as well as off-axis detector penetration. Since the overall resolution is a convolution of these factors, improvement of the positron range in conjunction with advances in the state-of-the-art detector technology can enhance the PET spatial resolution significantly^6^. Improving the spatial resolution of PET images would improve diagnostic accuracy and would also improve emerging cancer treatment strategies using theranostic radiopharmaceuticals (isotopes delivering both therapeutic and diagnostic benefits). Here we report, for the first time, on the impact of nanoscale magnetization by superparamagnetic iron oxide nanoparticles (SPIONs) on the range of positrons in PET imaging. Using PET-MRI, a dual-mode technology combining PET with Magnetic Resonance Imaging (MRI), we found that SPIONs labeled with a PET tracer can localize positron range by their strong nanoscale magnetization, producing images with noticeably improved spatial resolution.

**Figure 1:**
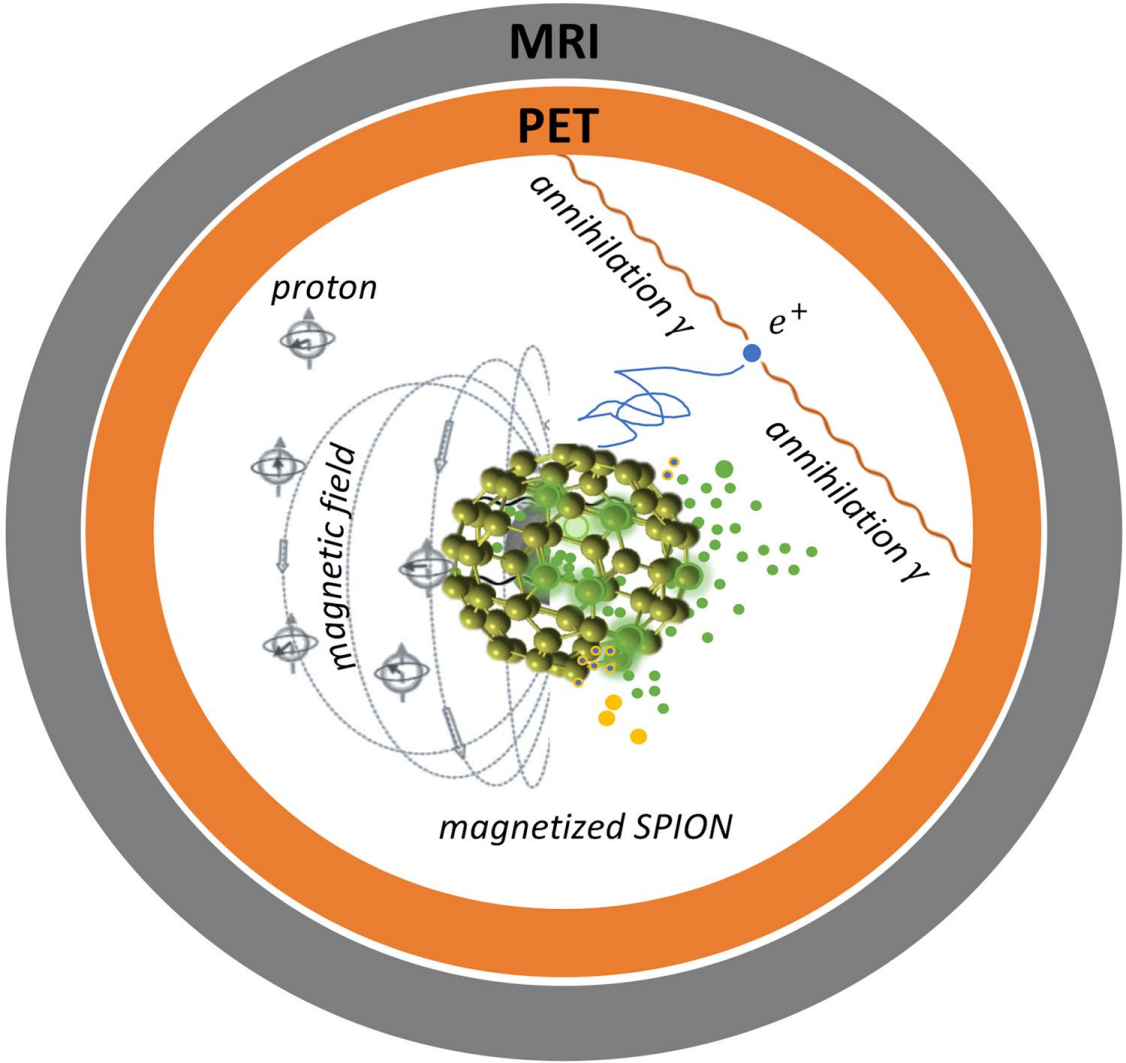
Schematic diagram illustrating the use of a radiolabeled magnetized superparamagnetic iron oxide nanoparticle for simultaneous multimodal imaging (*MRI* magnetic resonance imaging, *PET* positron emission tomography).

Depending on the parent nucleus, emitted positrons can have mean and endpoint energies ranging from 0.3 meV and 0.6 meV (for the most commonly used tracer, ^18^F) to 1.5 meV and 3.35 meV (for ^82^Rb), respectively. This corresponds to mean and maximum positron ranges of 2.4 and 17 mm, and 0.6 and 7.1 mm in tissue, with values in between for other commonly used isotopes (e.g. ^89^Zr, ^68^Ga, ^11^C)^7,8^. After thermalization, annihilation with an electron can occur either instantaneously, or can be delayed by the formation of a meta-stable intermediate positronium ($e^{ + } e^{ - }$) state^9^. In PET, coincidence detection of counter-propagating 511 keV annihilation photon pairs enables reconstruction of a three-dimensional (3D) image of the source region. The position of where these annihilation photons are created is different from the position of the parent nucleus, resulting in image blurring^1,3^. Recently, we showed that by delivering the PET tracer using SPIONs, PET-MRI technology can be leveraged to achieve better overall image quality by integrating the high sensitivity of PET with the high spatial resolution of MRI^10^ (see Fig. 1). In that study, the strong local magnetization of SPIONs enhanced MRI contrast by shortening the transverse (spin–spin) relaxation time of protons in surrounding water molecules. Here, we show that the strong nanoscale magnetization of SPIONs labeled with a PET tracer can also improve PET spatial resolution by localizing positron range and hence, the distribution of annihilation photons.

**Figure 2:**
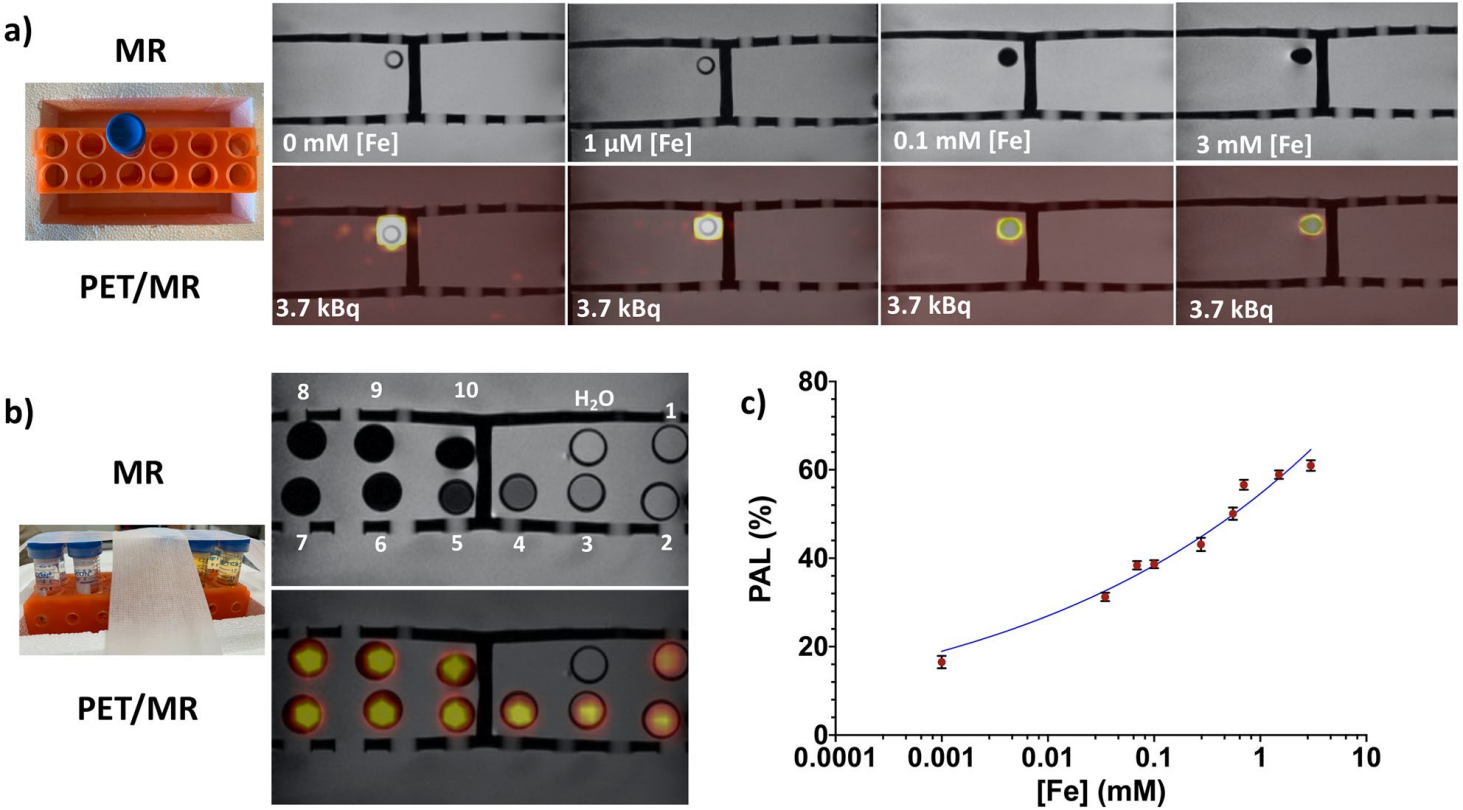
MR and co-registered PET/MR images. (**a**) Top and bottom panels show close-ups of T2*W MR and PET/MR phantom images for four of the ^89^Zr-SPION [Fe] concentrations, respectively. (**b**) MR and PET/MR images for all ten ^89^Zr-SPION [Fe] concentrations (listed in Table 1). (**c**) Plot of positron annihilation localization (PAL) as a function of [Fe].

A series of [Fe] dilutions (i.e. 0.001–3 mM, see Table 1) of ^89^Zr-SPION samples was prepared in 10 separate phantom vials (including ^89^Zr-water). The activity of ^89^Zr (half-life 74.4 days) in each phantom was kept constant, A_0_ = 3.7 kBq. Phantoms were scanned using a clinical PET-MRI scanner and images are shown in Fig. 2a,b. The top and bottom panels in Fig. 2a show individual close-ups of the T2*-Weighted (T2*-W) MRI and co-registered PET-MRI phantom images for four ^89^Zr-SPION [Fe] concentrations, respectively. Figure 2b shows images for all [Fe] SPION concentrations. MRI images of phantoms containing ^89^Zr-water (i.e. Fig. 2b phantom #1 0 mM [Fe]) and 1 µM [Fe] ^89^Zr-SPION (Fig. 2b phantom #2) did not show contrast against background water. However, at [Fe] = 0.1 mM (Fig. 2b phantom #5), the concentration was sufficient to cause strong dark contrast against water and at [Fe] = 3 mM, a geometric distortion of the image is discernable (Fig. 2b, phantom #10). This is due to a large magnetic susceptibility difference at the phantom boundary. The distortion is also evident in the PET-MRI images at [Fe] = 3 mM, indicating that the nanoscale amplification of magnetic field by the SPIONs restricts positron range. The integrated PET signal intensity for a circular region of interest for each phantom scan was calculated (see “Methods” section) to quantify this positron annihilation localization (PAL) effect (Fig. 2c).

**Table 1:** List of ^89^Zr-SPION [Fe] concentrations in each phantom and the percentage changes in the true and random counts for the phantom PET scans shown in Fig. 3a–d.

Phantom number	[Fe] mM	^89^Zr A_0_ (kBq)	PAL (%)	True (%)	Random (%)
1	0.000	3.70	0.00		
2	0.001	3.70	16.52	0.49 ± 0.04	5.10 ± 0.02
3	0.034	3.70	31.24		
4	0.069	3.70	38.40		
5	0.100	3.70	38.64	0.82 ± 0.04	6.00 ± 0.02
6	0.277	3.70	43.12		
7	0.553	3.70	50.09		
8	0.700	3.70	56.61		
9	1.500	3.70	58.90		
10	3.000	3.70	60.96	1.00 ± 0.05	6.30 ± 0.02

Although the lowest PAL occurred at [Fe] = 1 µM where SPION magnetization was insufficient to produce an obvious negative contrast against the background water, a ≈ 16% PAL was still observed. The nanoscale magnetic field gradient induced by SPIONs is strongly depended on its saturation magnetization and magnetic properties^11–14^. For instance, SPIONs in the low 1 µM [Fe] solution phantom (containing ≈ 2 × 10^15^ SPIONs with minimum SPION separation core-to-core distance of approximately 20 nm^15^) in an external 3 T MR magnetic field, can have an induced three dimensional (3D) local magnetic field around the SPION as high as 3 T at the SPION center^16–18^. Thus, an emitted $e^{ + }$ from the ^89^Zr atom at the surface of a radiolabeled ^89^Zr-SPION is influenced by not only the magnetic force (i.e. Lorentz force) from the magnetized SPION^16^ that it is labeled to but also the magnetic force of the other 2 × 10^15^ SPIONs in its vicinity. Collisions are suppressed in directions transverse to the local magnetic field. Therefore, compared to the ^89^Zr-water only phantom (i.e. the 0 mM [Fe] sample) shown in Fig. 2, phantoms with increasing magnetized ^89^Zr- SPION [Fe] concentrations exhibit PET signals that become increasingly spatially localized. Note this is different from the case of the Lorentz force exerted by the static magnetic field *B*_0_ of the MR magnet, which can restrict positron range in the transaxial direction, as has been reported in previous PET-MRI studies^19,20^. In this study, PAL is defined [cf. Eq. (1) in “Methods” section] relative to the PET signal when SPIONs are absent and thus measures the effect solely due to SPION magnetization. As demonstrated in Fig. 2c, PAL increased with [Fe] concentration of the ^89^Zr-SPIONs. Interestingly, our results show a significant PAL (≈ 40%) at a clinically relevant dose of 0.1 mM [Fe]^21^. As the presence of SPIONs increases mass density only by approximately 0.015%, the effect of collisions on positron range is negligible and thus, the observed PAL can be attributed to SPION nanoscale magnetization.

**Figure 3:**
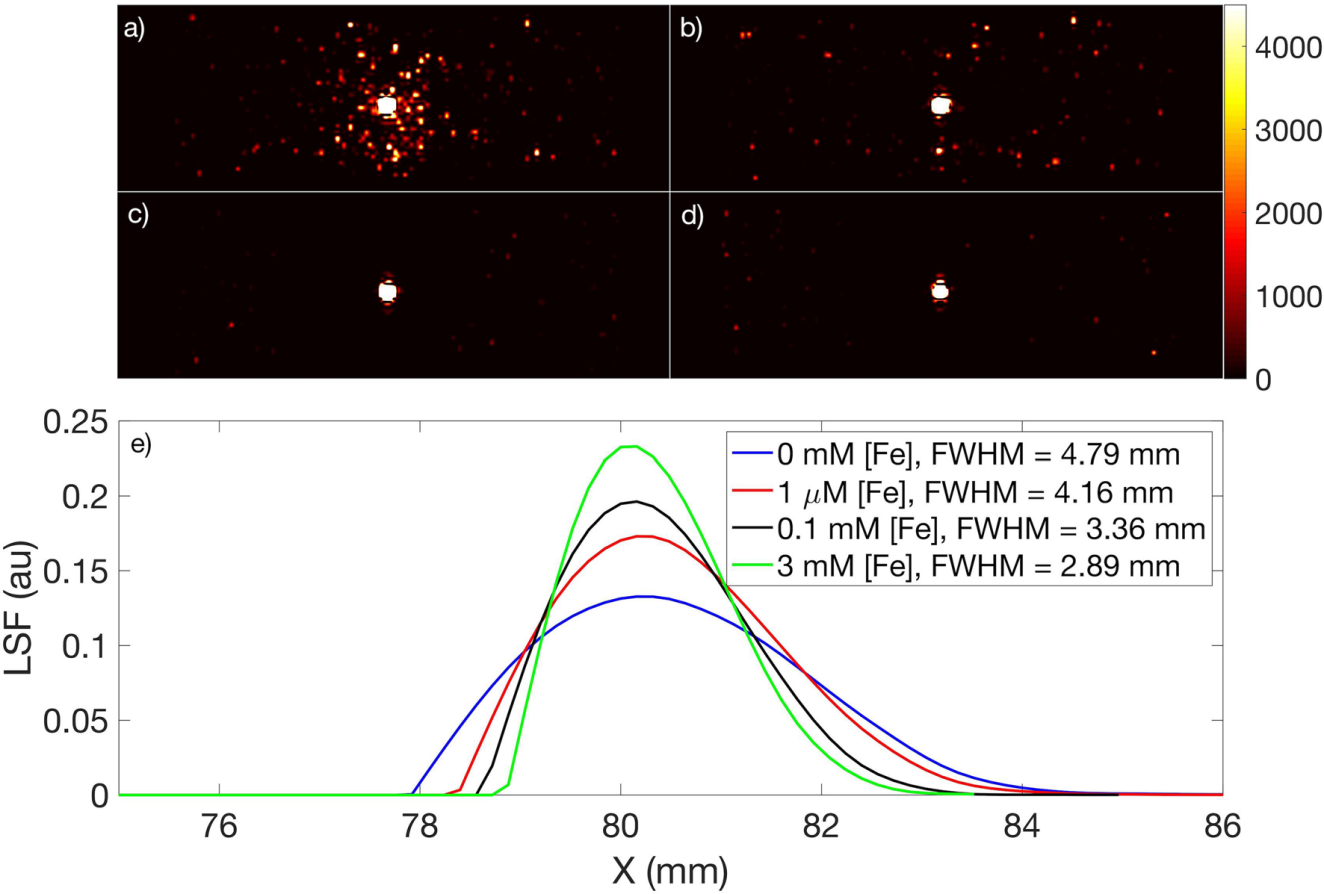
PET scans (axial view) of four vials containing ^89^Zr and varying amounts of SPIONs. (**a**) ^89^Zr only (i.e. 0 mM [Fe]) in de-ionized water. (**b**–**d**) ^89^Zr-SPION disperssion in de-ionized water with [Fe] = 0.001 mM, 0.1 mM and 3 mM, respectively. (**e**) The full width at half maximum (FWHM) of the line spread function (LSF) calculated for each PET scan to assess the impact of magnetized SPIONs on spatial resolution. The activity of ^89^Zr in each phantom was kept constant, A_0_ = 3.7 kBq.

Positron range restriction was further quantified by measuring the full width at half maximum (FWHM) of the PET images of the phantom vials with ^89^Zr in water and with ^89^Zr-SPIONs, with [Fe] = 0.001 mM, 0.1 mM and 3 mM (see Fig. 3a–d). These images show the distribution of positron annihilation events in water projected onto a plane (i.e. an integration of events). In comparison to the ^89^Zr in water image (Fig. 3a), it is evident that magnetized SPIONs increasingly localize the positron annihilation events within their vials as [Fe] increases. This is quantified with the FWHM of each line spread function (LSF, see “Methods” section) in Fig. 3e, which shows that spatial resolution improves by up to 40% in the presence of SPIONs. This can be attributed to the restriction of positron range by the strong nanoscale localization of magnetic field induced around the SPIONs in the presence of the external *B*_0_ = 3 T in the PET-MRI scanner. These results suggest that SPIONs radiolabelled with suitable PET tracers^10,15,22^ offer a novel approach to mitigating the well-known effect of positron range on spatial resolution in PET images^1,3–5^. Thus, in PET-MRI, SPIONs can not only enhance MRI contrast, but can also enhance PET spatial resolution, with the potential to improve overall image quality compared to that achieved with either modality separately.

**Figure 4:**
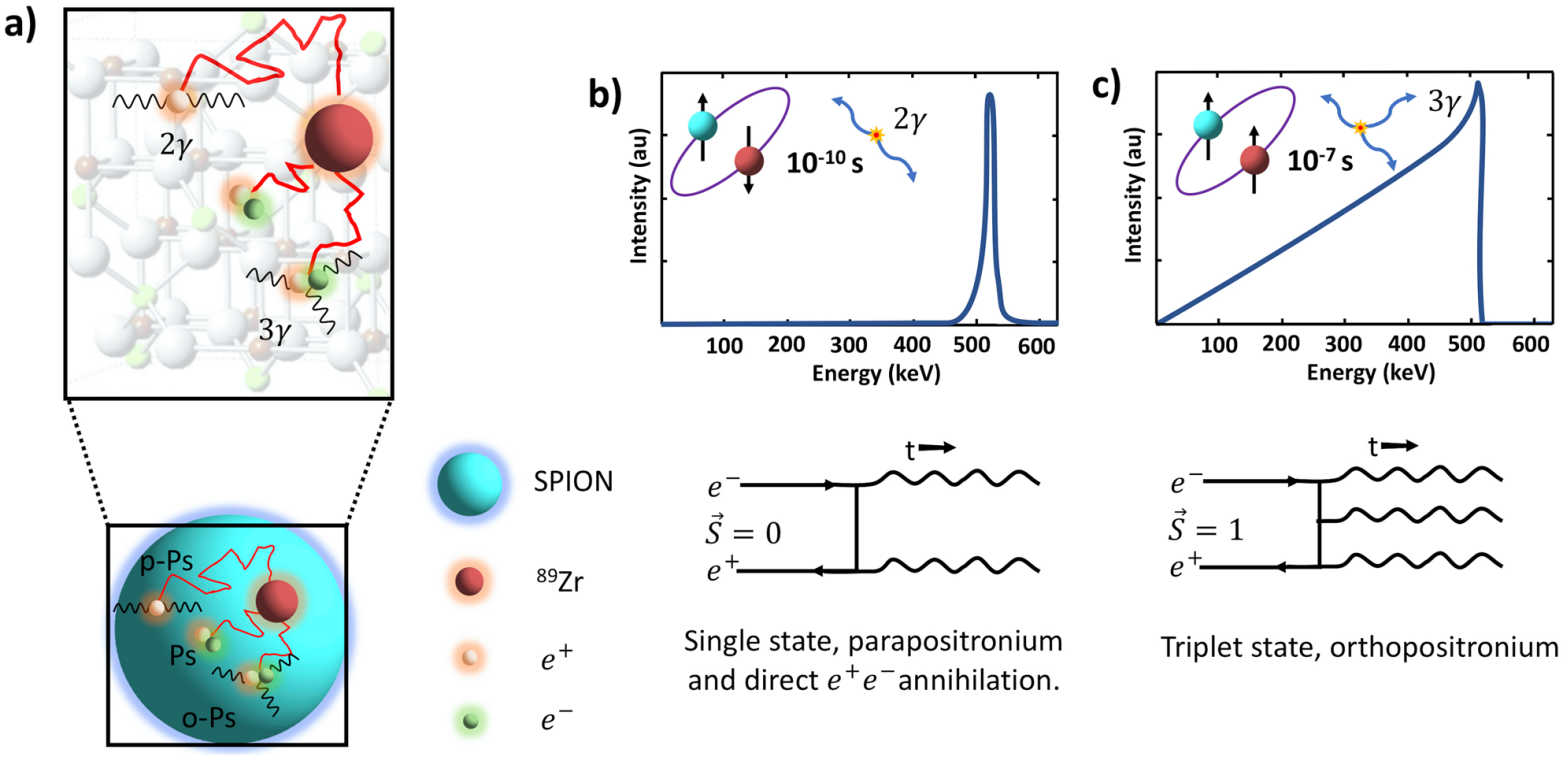
Schematic illustration of possible interactions of emitted $e^{ + }$ with radiolabeled SPIONs in water. (**a**) Different routes of positron annihilation: instantaneous annihilation with an electron in the surrounding water, formation of positronium (Ps) in the open volume defects within the SPION core Fe_3_O_4_ crystal structure or in surrounding water with two possible quantum states: the singlet state called para-Ps (p-Ps) decaying into two gammas and the triplet state called ortho-Ps (o-Ps) decaying in three gammas. (**b**,**c**) Corresponding schematic photon spectra: two back-to-back photons with equal energy of 511 keV for p-Ps decay and three annihilation photons with energies between 0 and 511 keV summing to 1022 keV for o-Ps respectively. Feynman diagrams for both p-Ps and o-Ps decay are illustrated beneath each spectrum.

We also found an increase of up to ≈ 1% and 6% in the true and random counts, respectively, detected by the PET scanner in phantom vials containing ^89^Zr-SPIONs compared to ^89^Zr only (see Table 1). This can be attributed to the interaction of positrons within the Fe_3_O_4_ crystal structure of SPION cores resulting in annihilation via formation of ortho-positronium (o-Ps, in the triplet state, ^3^S_1_) and emission of three gammas^23^. Such triple-coincidences may be processed as a set of double coincidence events or detected as random counts by the PET scanner^24^. However, further investigation is required for more accurate quantification of o-Ps and para-positronium (p-Ps) productions in SPION dispersions. Since most of the clinical and pre-clinical PET scanners are not capable of detecting o-Ps as triplet coincidences, positron annihilation lifetime spectroscopy might be the best option for accurately measuring the o-Ps yield in SPION dispersions^23^. Figure 4a is a schematic diagram demonstrating the possible interactions of the emitted $e^{ + }$ with the radiolabeled SPIONs in water. PET is based on registration of two gamma quanta originating from positron annihilation in tissue, where the most common (≈ 70%) annihilation route for a thermalized $e^{ + }$ is direct annihilation with an $e^{ - }$ (see Fig. 4b). The alternate route is via formation and decay of positronium (Ps)^25^. Ps can decay electromagnetically into two, three or more gamma quanta depending on the Ps quantum mechanical state just prior to annihilation^25^. Thus the energy of three annihilation photons has a continuum spectrum (with energies between 0 and 511 keV, with sum 1022 keV), due to energy and momentum conservation^26^ (see Fig. 4c). Due to spin statistics, 25% of all Ps are formed in the ground state (^1^S_0_) called para-positronium (p-Ps), while the remaining 75% form the triplet state (^3^S_1_), called ortho-positronium (o-Ps). In vacuum, o-Ps can only decay into at least three photons, and its lifetime (142 ns) is much longer than that of p-Ps (125 ps) or that of a free positron^25,27^. In water, due to the excess of oxygen atoms, the majority of o-Ps spins are inverted and thus the effective yield of annihilation quanta into three gammas approaches ≈ 1%^28^. The formation of o-Ps and the 3γ/2γ yield in materials can be significantly affected by open volume defects such as in the Fe_3_O_4_ crystal structure of SPION cores^23,29^. The lack of collinearity of the annihilation photons due to residual momentum of the $e^{ + }$ or Ps at the time of decay can also impact the spatial resolution in PET imaging. A previous study^30^ showed that p-Ps decays in solution have significantly more Doppler broadening of the annihilation photons compared to o-Ps and thus significantly higher residual momentum in p-Ps at the time of decay. This indicates that noncollinearity of annihilation photons in our study is mainly contributed by p-Ps. In our experiment, the true and random counts measured for the four phantoms (Fig. 3a–d) increased with increasing [Fe] concentration. This suggests the increase in true and random counts (listed in Table 1) may be attributed to the interaction of $e^{ + }$ with the crystal structure of SPION cores resulting in annihilation via formation of o-Ps (in the triplet state, ^3^S_1_) and decay into three gammas. Such triple-coincidences may be processed as a set of double coincidence events or a random event by the PET scanner, thereby incorrectly increasing the true and random counts detected by the PET scanner. Furthermore, the random counts recorded by the scanner for [Fe] = 3 mM is within 0.05% of the counts expected for a 6% increase in count rate relative to the [Fe] = 0 measurement. Furthermore, scintillators currently used in PET scanners, with an energy resolution that is typically worse than 15% at 662 keV and 35% at 340 keV, have low efficiency and sensitivity for detecting three annihilation photons (with a continuum spectrum)^31^. Nevertheless, triple coincidences may be identified by operating the scanner in LIST mode^32^. Another study has shown, with modifications in the acquisition electronics of a pre-clinical PET scanner, detection and processing of triple coincidences^32^. It would be interesting to follow up our study using emerging total body PET scanners, which with their superior sensitivity and 4π geometry are ideal for triple coincidence detection^33^. The novel Jagiel-lonian PET (J-PET) system^9,28,34^ has also been designed for detecting three gamma decay from ortho-positronium (o-Ps). Furthermore, a recent study has demonstrated a proof of principle of positronium imaging using combined Compton-PET imaging^35^. Furthermore, although pre-clinical PET-MR scanners would be suitable for future studies, it requires a custom-designed phantom suitable for radiolabeled SPIONs. For the targeted SPION concentrations and magnetization effect in this study, the volume and size of a capillary tube phantom (internal diameter < 1 mm with up to 250 µL^36^) would not be appropriate, as such a small volume could cause SPION aggregation, clumping and precipitation which can significantly impact measurement accuracy.

These results may also have implications for ongoing developments in cancer theranostics, where radiopharmaceuticals are used to both diagnose and treat cancer. Isotopes such as ^90^Y and ^64,67^Cu that are already in clinical use for PET imaging and radionuclide therapy (due to their $e^{ + }$ and $e^{ - }$ decays) could be administered by labelling, along with a tumor-targeting agent, onto SPIONs to take advantage of magnetization localization using a PET-MRI scanner. In this context, localization of charged particles by SPION magnetization could help to localize radiation dose within tumor cells, thereby enhancing therapeutic efficacy of these emerging cancer theranostic strategies^37–40^.

Overall, these results reveal a unique synergy between nanotechnology and medical imaging technology not previously recognized. In PET-MRI, the magnetic field induces a strong magnetization in SPIONs, which arises from their nanoscale geometric confinement. This nanoscale magnetization localizes the positrons emitted by the PET radiopharmaceutical, thus mitigating the fundamental limit imposed by finite positron range on PET image accuracy in a way that could not be achieved by any other means. As PET-MRI becomes increasingly more mainstream and SPIONs become an attractive alternative to existing MRI contrast agents, we anticipate further development and clinical translation of these synergistic 21st-century technologies.

## Methods

### SPIONs (Feraheme) were radiolabeled with ^89^Zr according to the published protocol^15,22^

Ten phantom vials were prepared with the following dimensions: diameter 17 mm; height 120 mm; and volume 15 mL (see Fig. 2). A series of dilutions of ^89^Zr-SPION samples were prepared in these 11 separate phantom vials with deionized water. A control phantom vial was made with ^89^Zr in deionized water only (phantom 1 in Fig. 2). All the phantoms were placed into a tube holder used for simultaneous PET-MRI. Phantom were then scanned using a simultaneous clinical PET-MRI scanner (3 T Biograph mMR). A head PET-MRI coil was used for both the PET and MRI scans. The PET scan was acquired over a period of 10 min. For each scan, the following settings were applied: scanner quantification factor = 88.9 M, branching factor = 0.228, phantom position = Head First-Supine, coincidence window width = 5.86 ns and an energy window with lower and higher levels of 410 and 610 keV respectively. Attenuation and scatter corrections were applied to all the images, which were reconstructed from the PET data using an iterative reconstruction algorithm (Ordered Subset Expectation Maximization (OSEM) with 3 and 21 iterations and subsets respectively). Multi-slice T2W, T1W and T2*W images were acquired using a turbo spin echo (TSE) sequence. Multiple acquisitions of the T1W (at TE = 11 ms with TR = 350 ms) and T2W (at TE = 90 ms with TR = 3000 ms) and T2*W (at TE = 9 ms with TR = 350 ms) scans were acquired. The mean magnitudes of PET image signal intensities were obtained within drawn circular regions of interest (ROIs) using MATLAB software for each sample to calculate the PAL for each phantom according to Eq. (1):

1 $${\text{PAL}} = \left( {{\text{SER}} - 1} \right) \times 100$$

where SER is the signal enhancement ratio (i.e. PET signal intensity image with SPIONs/PET signal intensity image without SPIONs) within the ROI. To assess the spatial resolution of the PET images, the FWHM was calculated from the line spread function (LSF) based on the edge smearing function (ESF) using Eqs. (2) and (3)^10^:

2 $$LSF = \frac{d }{{dx}}ESF\left( x \right)$$

3 $$ESF = \frac{{X - \frac{1}{n}\mathop \sum \nolimits_{i = 1}^{n} x_{i} }}{{\sqrt {\frac{1}{n}\mathop \sum \nolimits_{i = 1}^{n} \left( {x_{i} - \overline{x}} \right)^{2} } }}$$

The *ESF* was calculated from line profiles of the PET images, where *X*,* x*_*i*_, and *n* are the input array of voxel intensity data, the *i*th element of* X* and the number of voxels used in averaging, respectively.

## Data Availability

Data are available upon reasonable request.
